# Virulence perspective genomic research unlocks the secrets of *Rhizoctonia solani* associated with banded sheath blight in Barnyard Millet (*Echinochloa frumentacea*)

**DOI:** 10.3389/fpls.2024.1457912

**Published:** 2024-10-28

**Authors:** T. S. S. K. Patro, K. B. Palanna, B. Jeevan, Pallavi Tatineni, T. Tharana Poonacha, Farooq Khan, G. V. Ramesh, Anusha M. Nayak, Boda Praveen, M. Divya, N. Anuradha, Y. Sandhya Rani, T. E. Nagaraja, R. Madhusudhana, C. Tara Satyavathi, S. Koti Prasanna

**Affiliations:** ^1^ Agricultural Research Station, Acharya N. G. Ranga (ANGR) Agricultural University, Vizianagaram, Andhra Pradesh, India; ^2^ ICAR-All India Coordinated Research Project (ICAR-AICRP) on Small Millets, Project Coordinating (PC) Unit, University of Agricultural Sciences, Bengaluru, Karnataka, India; ^3^ Crop Protection Division, ICAR-National Rice Research Institute, Cuttack, Odisha, India; ^4^ Department of Plant Pathology, University of Agricultural Sciences, Bengaluru, Karnataka, India; ^5^ Department of Plant Pathology, Punjab Agricultural University, Ludhiana, Punjab, India; ^6^ ICAR- Indian Institute of Millets Research, Hyderabad, Telangana, India; ^7^ Department of Plant Biotechnology, University of Agricultural Sciences, Bengaluru, Karnataka, India

**Keywords:** banded sheath blight, genomics, mitochondrial genome, secretome, cutinase protein, *Rhizoctonia solani*

## Abstract

**Introduction:**

Banded sheath blight (Bsb) disease, caused by *Rhizoctonia solani*, is an emerging problem in barnyard millet cultivation. One of the significant goals of pathogenomic research is to identify genes responsible for pathogenicity in the fungus.

**Methods:**

A virulence profiling-based approach was employed and six *R. solani* isolates were collected from various ecological zones of India. The morphological parameters and virulence of all of the six *R. solani* isolates were investigated. The most virulent strain was designated as RAP2 and its genome has been sequenced, assembled, and annotated.

**Results:**

The RAP2 genome is 43.63 megabases in size and comprises 10.95% repetitive DNA, within which 46% are retroelements, 8% are DNA transposons, and 46% are unidentified DNA. The Gene Ontology (GO) annotation of RAP2 proteins revealed that “phosphorylation”, “membrane”, and “ATP binding” have the highest gene enrichment in the “biological process”, “cellular component” and “molecular function” domains, respectively. The genome comprises a majority of secretory proteins in the pectin lyase fold/virulence factor superfamily, which break down plant cell wall polymers to extract saccharides. The RAP2 genome is comparable to *R. solani*, which infects maize and rice, but it diverges further from soybean in terms of nucleotide-level genetic similarity. Orthologous clustering of RAP2 protein sequences with *R. solani* infecting maize, rice, and soybean yields 5606 proteins shared across all genomes. GO analysis of 25 proteins specific to the RAP2 genome found enrichment in the ethylene response, which can cause spore germination and infection in host plants.

**Discussion:**

Interestingly, a 28-bp deletion in the RAP2 strain’s cutinase domain was discovered in the cutinase protein, which might be important in the infection process, perhaps rendering the enzyme inactive or allowing the pathogen to infect barnyard millet while avoiding host defense. This study sheds light on the genetic makeup of *R. solani*, allowing researchers to discover critical genes related with pathogenicity as well as potential targets for fungicide development.

## Introduction

*Rhizoctonia solani* is a destructive soil-borne, necrotic fungal pathogen. Its sexual stage, *Thanatephorus cucumeris* (A.B. Frank) Donk, belongs to the division Basidiomycota. *R. solani* is a pathogen with a wide host range, infecting a wide variety of plant species, particularly economically significant crops like rice, soybean, potato, sugar beetroot, canola, cotton, lettuce, coffee, etc (Chahal et al., 2003; Ajayi-Oyetunde and Bradley, 2018). Furthermore, because *R. solani* may live for several seasons and spread through sclerotia in the soil, it is a difficult pathogen to eradicate (Hoitink et al., 1991).

Morphological characteristics have generally been utilized to determine variability among fungal isolates. However, because they lack distinguishing morphological features, *Rhizoctonia* species are not well understood (Vilgalys and Cubeta, 1994). *R. solani* isolates have been classified into 14 anastomosis groups (AG1 to AG13 and AGB1) based on the hyphal fusion reactions (Carling et al., 2002; Singh et al., 2019). *R. solani* is a large species complex thought to consist of many genetically distinct groups with a wide range of life histories, most likely containing numerous species (Anderson, 1984; Vilgalys and Cubeta, 1994; Cubeta and Vilgalys, 1997). Moreover, based on sexual phases, the groups cannot be separated since a large number of the field isolates do not sporulate in a laboratory conditions. Accordingly, scientists recognize *R. solani* as a single species and further categorize it into anastomosis groups (AGs) and subgroups within AGs (Ogoshi, 1987).

Recently, most small millets have experienced a severe loss in grain output due to banded sheath blight (Bsb) disease, caused by *R. solani* and is particularly problematic in hot and humid climate regions of India (Patro et al., 2021). The earliest known food grains from the Poaceae family that have been traditionally cultivated and consumed by humans are small millets. These crops are typically grown on marginal land and in rain-fed locations by the majority of resource-poor farmers in Asia and African countries (Changmei and Dorothy, 2014; Palanna et al., 2023). Finger millet [*Eleusine coracana* (L.)], Foxtail millet (*Setaria italica*), Little millet (*Panicum sumatrense*), Kodo millet (*Paspalum scrobiculatum* L.), Barnyard millet (*Echinochloa frumentacea*), Proso millet (*Panicum miliaceum* L.), and Browntop millet (*Brachiaria ramosa* L.) are the most commonly grown small millets.

India is the world leader in small millet production, with an output of 3.46 lakh metric tons and a productivity of 7.81 q/ha (Nagaraja et al., 2023). In India, barnyard millet cultivation is the second most significant small millet after finger millet (Padulosi et al., 2009). Although barnyard millet is resilient to many adverse environmental circumstances, there’s evidence that the severity of Bsb disease has increased in recent years, which has led to a significant decrease in grain (44%) and fodder (36.49%) yield (Palanna et al., 2024). To establish effective management strategies, a thorough understanding of diversity, virulence, disease epidemiology, and pathogenomics is essential. Previous studies have shed light on the prevalence of the disease, different approaches to management, resistant cultivars, and diagnostics (Patro et al., 2020; Patro et al., 2021; Rawat et al., 2022). However, little or no information is available about the diversity and pathogenomics of *R. solani* that cause Bsb in barnyard millet. Therefore, the current study is the first to offer a comprehensive overview of *R. solani* on barnyard millet, with the following objectives in mind: (1) Survey, morphological, and virulence characterization of fungal isolates; and (2) Genome sequencing, assembly, and analysis of a highly virulent strain of the pathogen *R. solani*.

## Materials and methods

### Field survey, collection, isolation, and purification of *Rhizoctonia* isolates

In this study, *Rhizoctonia* isolates were obtained from infected tissues of barnyard millet plants exhibiting Bsb symptoms during *Kharif* 2021 from different ecological zones of India, including coastal belts, hilly ecosystems, and northern plains (**Table 1**). After being cut into small pieces, the infected samples were surface sterilized with 70% (v/v) ethyl alcohol, suspended in mercuric chloride (HgCl_2_) for 30 seconds, and then rinsed with sterilized distilled water. The surface sterilized samples were dried and transferred to potato dextrose agar (PDA) media containing petri dishes amended with 500 ppm streptomycin sulfate and maintained at 27 ± 1°C in an incubator.

**Table 1:** List of isolates collected from different barnyard millet growing regions of India.

Code of the isolate	Place of Collection	State
RAP -1	Hiramandalam	Andhra Pradesh
RAP -2	Vizianagaram	
ROD -1	Brahmapura	Odisha
ROD -2	Semiliguda	
RUK -1	Ranichauri	Uttarakhand
RMP -1	Rewa	Madhya Pradesh

### Morphological variability of *Rhizoctonia* isolates

The study analyzed six isolates’ pure cultures, recording morphological characteristics like colony diameter, color, appearance, and hyphal width and sclerotial observations like color, size, number, texture, and pattern of colony formation.

### Pathogenicity and virulence analysis

Barnyard millet (LDR-1) seedlings were raised in earthen pots containing a 2:1 vol/vol ratio of autoclaved soil and vermicompost. Six isolates were assessed for pathogenicity using artificial inoculation on the one-month-old susceptible variety LDR-1 using the standard inoculation technique as reported by Shekhar and Kumar (2012). Seedlings were kept in a glasshouse at a temperature of 27 ± 1°C and a relative humidity of over 85%-90%. The experiment was conducted three times for each isolate, and it was repeated twice. Responses and percent disease index were documented 25 days’ post-inoculation using a standard assessment scale (Palanna and Das, 2022).

### DNA extraction, ITS gene sequencing, and phylogeny

**Figure 1:**
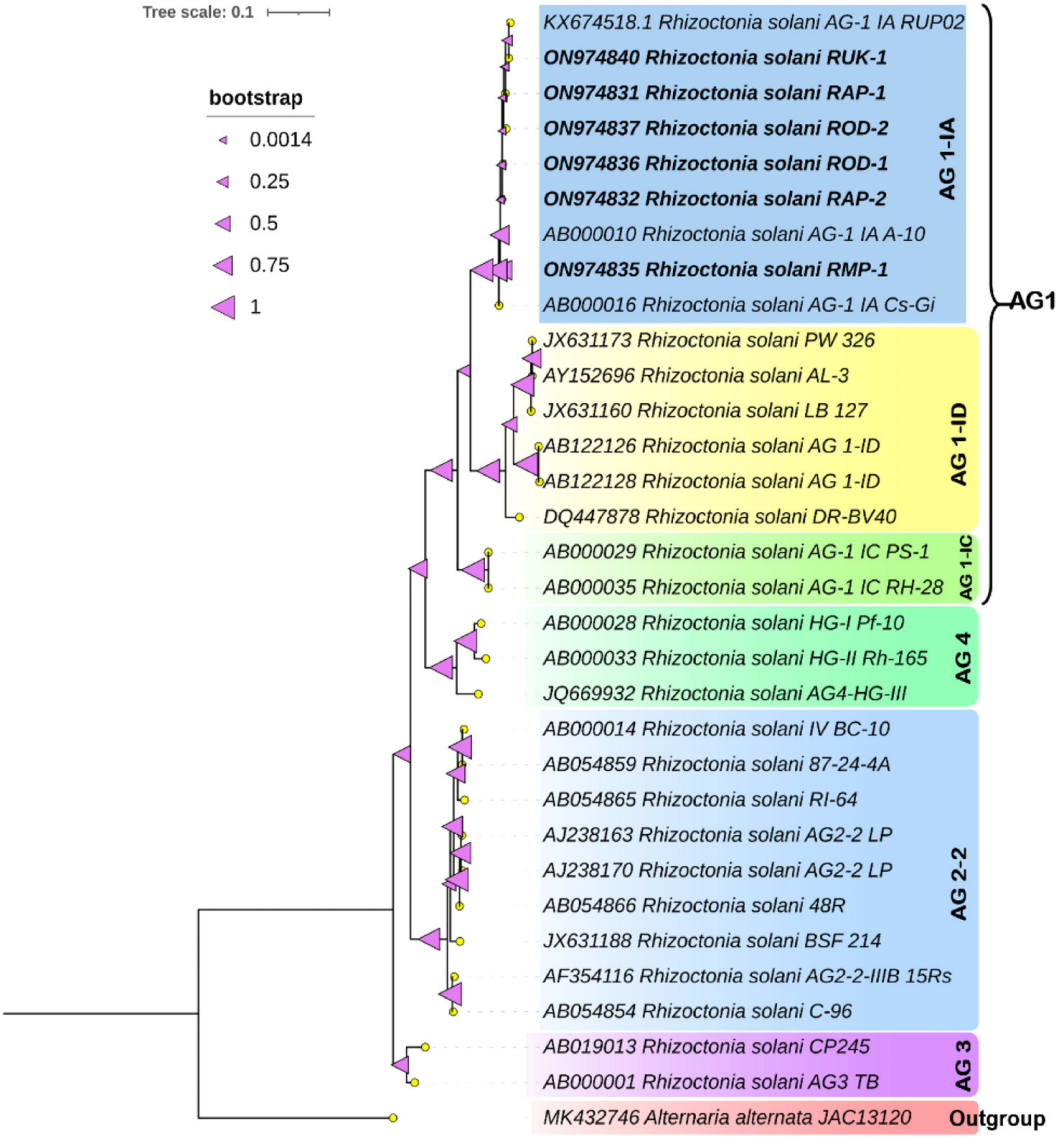
Phylogenetic tree of ITS region inferred from maximum-likelihood approach illustrating the genetic relationships among the R. solani isolates. The tree was constructed with 1000 bootstrap replicates using Alternaria alternata JAC13120 as an outgroup.

Genomic DNA was isolated from each of the six *Rhizoctonia* isolates using the Cetyl-Trimethyl Ammonium Bromide (CTAB) procedure (Murray and Thompson 1980). Genomic DNA was extracted and its yield and purity evaluated using a Thermo Fisher Scientific NanoDropTM 1,000 Spectrophotometer. For PCR amplification, the DNA concentration was adjusted to 50-100 ng/μl. PCR reactions were carried out in a thermal cycler (Eppendorf Mastercycler V ProTM) using 25µl of the reaction mix. It contained 50–100 ng of DNA template, 25 mM MgCl_2_, 2 mM of each dNTP, 1X Taq buffer, Taq DNA polymerase - 1U, and nuclease-free water. The internal transcribed spacer (ITS) gene fragment was amplified with primer pairs ITS1/ITS4. A five-minute initial denaturation at 95°C was followed by 35 cycles of denaturation for one minute at 95°C, primer annealing for one minute at 57°C, and extension for two minutes at 72°C. A final extension step was conducted for ten minutes at 72°C. Ethidium bromide-stained 1% agarose gel was used to resolve the amplicons. The PCR products were forwarded to Bengaluru-based Molsys Pvt. Ltd., a privately held company, for sequencing. In order to receive the accession numbers, the ITS sequences of all 6 isolates were also uploaded to NCBI GenBank (http://www.ncbi.nlm.nih.gov). Using MEGA-X software, a phylogenetic analysis of ITS genes belonging to six *Rhizoctonia* isolates and reference sequences of representative strains was performed with 1,000 bootstrap repetitions. The fungal strains utilized to build the phylogram are outlined completely in **Figure 1**.

### Genome sequencing, assembly and annotation

The DNA library was prepared using the TruSeq DNA Nano LP kit, quantified using the Qubit 4 Fluorometer, and sequenced on the Illumina Novaseq 6000 system to output 159 bp paired-end reads. The raw reads were assessed for quality using FastQC v. 0.11.9 (Andrews, 2019), and subjected to adapter removal and quality filtration by applying default settings in addition to the removal of 10 bases from the 5’ end and 5 bases from the 3’ end of the paired end reads using Trim Galore v.0.6.5 (Felix, 2022).

The quality-filtered reads were *de novo* assembled using SPAdes v. 3.15.5 (Bankevich et al., 2012) with default settings. The finished assembly file was subjected to genome-guided assembly correction and scaffolding using RagTag v.2.1.0 (Alonge et al., 2022) with the *Rhizoctonia solani* AG-1 IA strain RefSeq (GCF_016906535.1) genome as a reference. The assembly scaffold file was further cleaned using Funannotate v.1.8.7 (Palmer and Stajich, 2020) to remove repetitive contigs, and contigs with a minimum length of 500 bp were retained. The assembly quality statistics were evaluated using QUAST v.5.2.0 (Gurevich et al., 2013), and the assembly completeness was evaluated with BUSCO v.5.1.2 (Manni et al., 2021), using Agaricomycetes_odb10.

Gene prediction was carried out using the Funannotate v.1.8.7 pipeline’s ‘predict’ command using the *Rhizoctonia solani* AG-1 IA strain RefSeq (GCF_016906535.1) genome’s RNA sequences as transcript evidence. Predicted protein sequences were annotated using Uniprot proteins (The UniProt Consortium, 2023) by creating a local blastable database, followed by homology searches using protein blast (Altschul et al., 1990; Camacho et al., 2008).

### Repetitive landscape of RAP2 genome

Genome annotation began with the identification of repetitive elements. Isolate-specific repeats were identified *de novo* using RepeatModeler v.2.0.3 (Flynn et al., 2020) and annotated using Repbase release v.20181026 (Bao et al., 2015) libraries. RepeatMasker v.4.1.3-p2 (Tarailo-Graovac and Chen, 2009) was used to mask simple repeats, curate repeats from Repbase databases for the fungi, and isolate specific repeats. The genome was scanned for simple sequence repeat identification and classified using the online server MegaSSR (Morad et al., 2023).

### Mitochondrial genome

The RAP2 genome was searched for its mitochondrial genome by using the AG1-IA (GCF_016906535.1) mitochondrial genome sequence as a query by making a local blastn v.2.10.0+, and the best hit sequence with the highest percent identity and query coverage was identified. The RAP2 mitochondrial genome was then analyzed using blastn against the NCBI NR database to identify the closest mitochondrial genomes. Gene prediction and annotation were performed using the online version of MFannot (Lang et al., 2023), and the circular genome representation was performed using the online visualization tool GenomeVx (Conant and Wolfe, 2008).

### Pathway and gene ontology analysis

The RAP2 assembly protein sequences were analyzed using BlastKOALA (Kanehisa et al., 2016) to assign Kegg Orthology (KO) identifiers, and subsequently pathway annotations were performed using the KEGG Mapper analysis (Kanehisa and Sato, 2020; Kanehisa et al., 2022). UniprotR package v.2.3.o (Soudy et al., 2020) was used to query the GO terms to obtain the top 10 enriched GO terms related to biological processes, molecular function, and subcellular localization.

### Secretome analysis

For secretome prediction, the online Secretool (Cortázar et al., 2014) website was used to evaluate the protein sequences of the RAP2 genome. To further separate secreted proteins from secreted effectors, the predicted secretome proteins were submitted to the EffectorP 3.0 web service (Sperschneider and Dodds, 2022). Superfamily annotations were obtained by screening the secretome proteins against the InterPro protein signature databases (Paysan-Lafosse et al., 2022). Using the dbCAN3 (Jinfang et al., 2023) web server, the RAP2 genome was annotated for carbohydrate-active enzymes by means of the HMMER search for CAZyme family annotation against the dbCAN CAZyme domain HMM database and the HMMER search for CAZyme subfamily annotation against the dbCAN-sub HMM database of CAZyme subfamilies.

### Comparative genomics analysis

Genome of *Rhizoctonia solani* isolated from maize (GCF_016906535.1), and genomes of *Rhizoctonia solani* isolated from rice (GCA_015342405.1) and soybean (GCA_026686785.1) along with the assembled RAP2 genome were selected for the comparative genomics analysis. The genome fasta files were submitted to the MOSGA (Martin et al., 2020, 2021) web server for the comparative genomics analysis. Genome completeness and Average nucleotide identity were evaluated and plotted. The OrthoVenn3 (Sun et al., 2023) web server was used to identify orthologous gene clusters across genomes. With special interest in fungal cutinases, the RAP2 genome was scanned for the cutinase proteins and searched for the presence of the cutinase domain. The protein with the intact cutinase domain was again searched in the genomes selected for comparative genomics study using blastp, and the best hit sequences were subjected to multiple sequence alignment using the online Clustal Omega (Sievers and Higgins, 2018) web server.

## Results

### Isolation and morphological characterization

**Figure 2:**
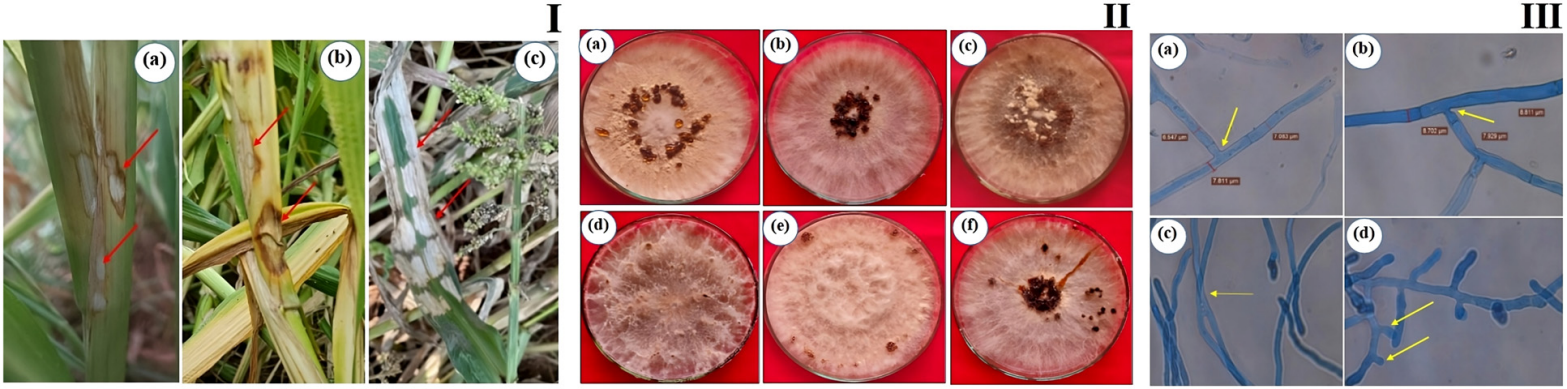
Sheath blight disease symptoms and morphological characteristics of *R. solani* isolates **(I)** Disease symptoms on barnyard millet caused by *R. solani*. **(a)** Initial symptoms on the stem, **(b)** Subsequent larger lesions, **(c)** Severe symptoms with characteristic banded appearance **(II)** Colony morphology of *Rhizoctonia* isolates **(a)** RAP 1, **(b)** RAP 2, **(c)** ROD 1, **(d)** ROD 2, **(e)** RUK 1, and **(f)** RMP 1; **(III)** Typical hyphal characteristics of *R. solani* **(a)** Right angle branching, **(b)** Constriction at the point of origin, **(c)** Hyphal fusion, and **(d)** Barrel shaped moniloid cells.

Bsb disease samples were collected from barnyard millet-infected fields and purified using the hyphal tip method, as per standard tissue isolation protocols. Six *Rhizoctonia* cultures were obtained, each with an identifying code (**Table 1**). The colonies were light yellow or brownish yellow, and they grew on PDA media for 5 days, measuring 84.6 mm to 90 mm. Hyphae branched at a right angle, and cottony mycelium grew profusely in regular or irregular patterns. Sclerotia ranged in size from 1.43 mm to 1.91 mm, were brown to dark brown, and might be rough or smooth. The distribution pattern consisted of a center, a sub-central ring, and a scattered, distributed, irregular, or peripheral ring (**Figure 2**). **Tables 2**, **3** summarize the morphological traits shared by the six *Rhizoctonia* isolates.

**Table 2:** Morphological characteristics of *Rhizoctonia* isolates.

Sl. No.	Isolate	Colony appearance	Colony colour	Colony diameter in mm (5 DAI)	Hyphal Width in μm
1	RAP-1	Profusely growing Cottony mycelium	Brownish Yellow	90.0	7.38
2	RAP-2	Profusely growing Cottony fluffy aerial mycelium	Light yellowish brown	90.0	4.57
3	ROD-1	Profusely growing Cottony fluffy aerial mycelium	Yellowish Brown	88.6	8.62
4	ROD-2	Profusely growing Cottony fluffy aerial mycelium	Yellowish brown	90.0	4.86
5	RUK-1	Profusely growing Cottony mycelium	Brownish Yellow	84.6	6.26
6	RMP-1	Profusely growing Cottony fluffy aerial mycelium	Yellowish brown	90.0	9.49

All parameters are considered after observing three replicates per isolate.

**Table 3:** Variability in sclerotial characteristics among different *Rhizoctonia* isolates of on PDA.

Sl. No.	Isolate	Colour of sclerotia	Size of sclerotia in mm	Sclerotial number	Sclerotial Texture	Sclerotial pattern
1	RAP-1	Dark Brown	1.87	35	Rough	Sub-central ring
2	RAP-2	Dark Brown	1.65	40	Rough	Centre
3	ROD-1	Brown	1.84	43	Smooth	Scattered
4	ROD-2	Dark Brown	1.43	39	Rough	Irregular
5	RUK-1	Dark Brown	1.67	12	Rough	Peripheral ring
6	RMP-1	Dark Brown	1.91	38	Rough	Centre and scattered

All parameters are considered after observing three replicates per isolate.

### Pathogenicity and virulence

The barnyard millet susceptible cultivar LDR-1’s basal leaf sheath was inoculated with PDA plugs containing actively growing mycelia of *R. solani* isolates. Each of the six isolates caused characteristic lesions on the leaf sheath four to eight days after inoculation. The virulence spectrum was assessed using a standard evaluation scale (Palanna and Das, 2022) on the PDI. The susceptible cultivar LDR-1 had typical lesions with PDI values ranging from 18.52 to 55.56. RUK1 isolate showed the lowest disease incidence, while RAP2 isolate had the highest PDI (**Table 4**).

**Table 4:** Virulence analysis of *Rhizoctonia* isolates on barnyard millet under glasshouse conditions.

Sl. No.	Isolate	No. of days for symptom expression	Percent Disease Index 25 days post inoculation
1	RAP-1	5	48.15
2	RAP-2	4	55.56
3	ROD-1	6	48.15
4	ROD-2	5	40.74
5	RUK-1	8	18.52
6	RMP-1	7	25.93

### Molecular characterization

Primer pairs produced clear bands of about 600 bp in all six isolates. After PCR product purification, sequencing, and curation, the sequences have been deposited at NCBI GenBank. A phylogenetic tree was constructed using the reference gene sequences with various AGs and the ITS sequences from six *Rhizoctonia* isolates. All the six *Rhizoctonia* isolates were grouped with the AG1-IA *Rhizoctonia* strains and clustered into a single clade with clear resolution among the different AG groups studied (**Figure 1**).

### Genome sequencing and assembly

The most virulent isolate, RAP2, has been selected for whole genome sequencing based on the virulence study. The majority of the reads sequenced had high-quality bases, and after filtration, 99.41% (**Supplementary Table S1**) were available for assembly. Following genome assembly, scaffolding, and sorting, 4162 contigs were assembled, yielding a 43.63 Mb genome. The longest contig was 3788558 bp (8.6% of the assembly), while the N50 and N90 contigs measured 1854826 and 3779 bp, respectively. Furthermore, the L50 and L90 values of the assembly were 8 and 664, respectively (**Supplementary Table S2**). When assessed against 2898 conserved profiles from the Agaricomycetes_odb10 ortholog lineage dataset, the genome assembly was found to be 78.6% complete, with 5.8% fragmented and 15.6% missing (**Supplementary Figure S1**). Gene prediction returned 9828 protein sequences, which were then annotated with 22082 proteins associated with “*Rhizoctonia solani*” species retrieved from the Uniprot database. A homology search of the 9828 proteins using blastp v.2.10.0+ against the “*Rhizoctonia solani*” database of 22082 proteins revealed that around 73% of the 9828 proteins had more than 90% homology with the proteins in the database (**Supplementary Table S3**).

**Figure 3:**
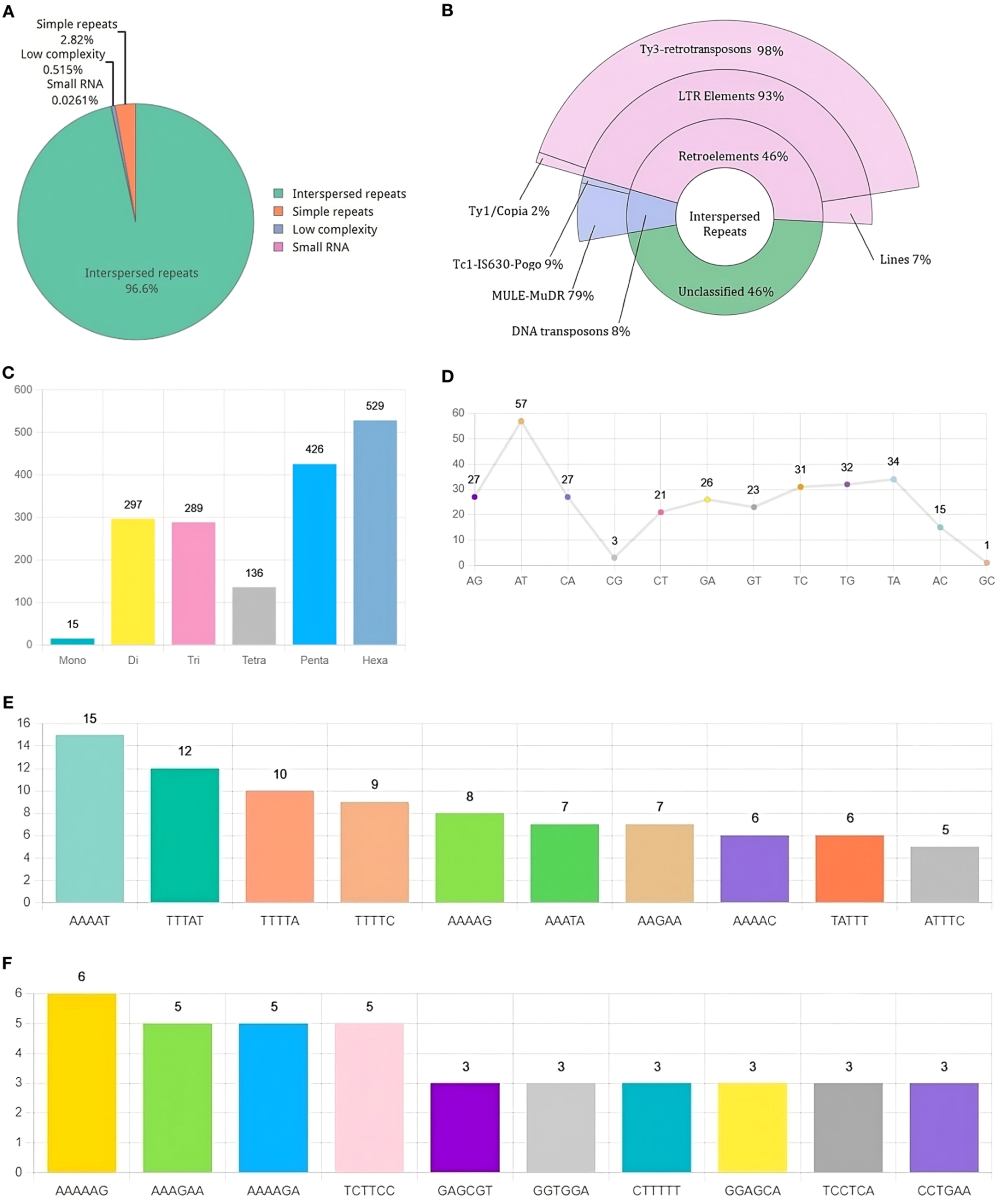
Landscape of Repetitive DNA in *R. solani* (RAP2) genome **(A)** Composition of repetitive DNA **(B)** Classification of interspersed repeats **(C)** Distribution of SSR classes **(D)** Distribution of Di-Nucleotide SSRs **(E, F)** Distribution of Penta and Hexa Nucleotide SSRs.

Repetitive DNA makes up 10.95% of the RAP2 genome, out of which 96.6% is made up of interspersed repeats (**Figure 3A**). Interspersed repeats are annotated as Retroelements (46%), DNA Transposons (8%), and unclassified (46%). LTR elements comprise 93% of the major retroelements, Ty3-retrotransposons make up 98% and Ty1/Copia elements make up just a minor portion (**Figure 3B**). Di, penta, and hexa nucleotide repeats are the main SSR classes found in the RAP2 genome (**Figure 3C**). The two primary dinucleotide SSRs in the genome were “AT” and “TA” (**Figure 3D**), while the penta and hexa SSRs were “AAAAT” (**Figure 3E**) and “AAAAAG” (**Figure 3F**).

**Figure 4:**
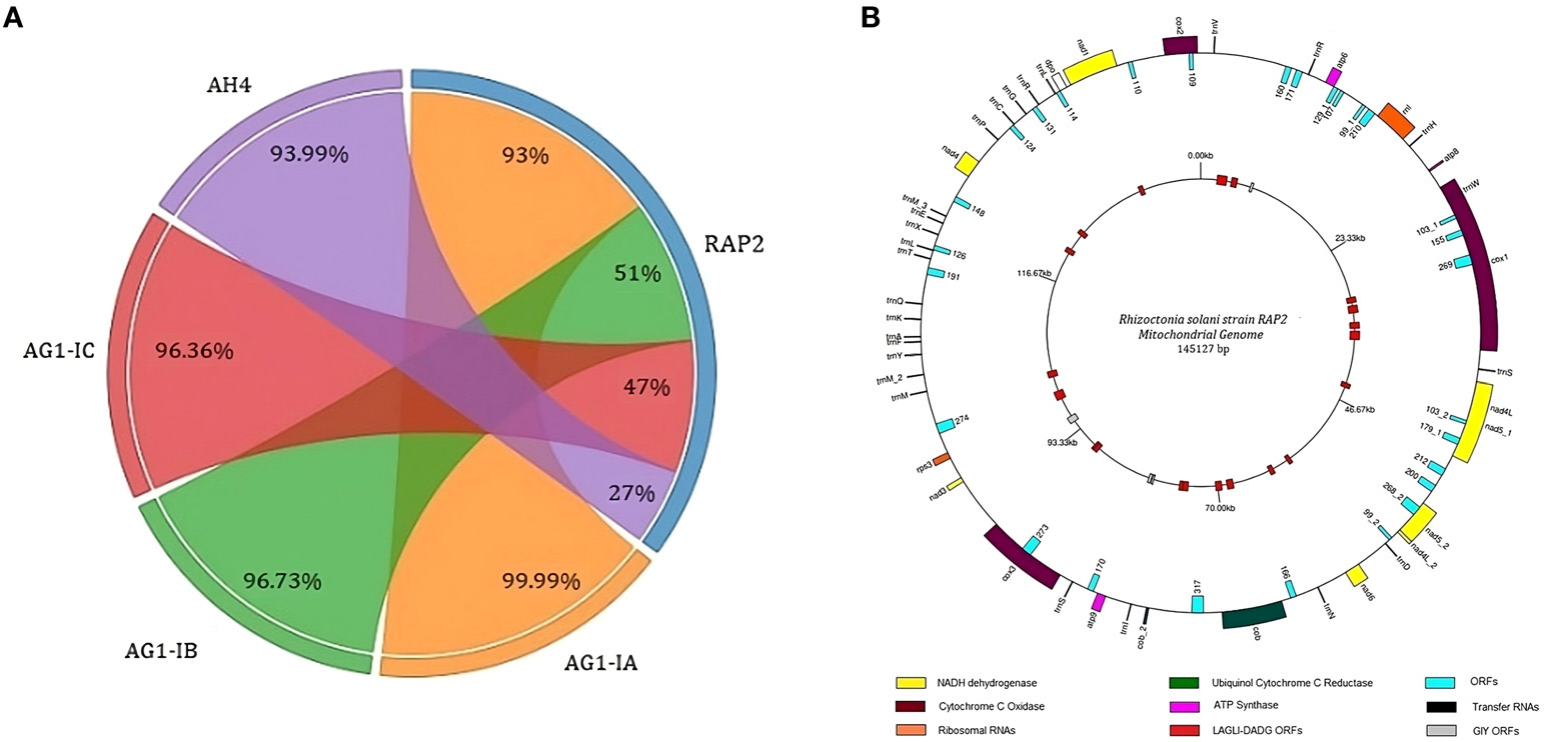
The structure of the mitochondrial genome of *Rhizoctonia solani* strain RAP2 **(A)** Chord diagram showing the Identity percentage and query coverage of RAP2 genome with AG1-IA, AG1-IB, AG1-IC and AH4 mitochondrial genomes. Weights on RAP2 chord correspond to the query converge percentage and corresponding weights on the AG1-IA, AG1-IB, AG1-IC and AH4 are identity percentages; **(B)** The outer circle of the genome structure houses the protein coding genes, tRNAs and ORFs. The inner circle consists of the ORFs related to GIY and LAGLI-DADG endonucleases.

A single contig was identified as the mitochondrial genome, measuring 145,127 bp in length. This contig exhibited 99.9% sequence identity with the AG1-IA mitochondrial genome and demonstrated a 93% sequence overlap (**Figure 4A**). Gene prediction revealed 99 protein-coding genes in the RAP2 mitochondrial genome. The genome included 26 mitochondrial tRNAs and 17 core genes of electron transport and oxidative phosphorylation; comprising two copies of cob, cox1, cox2, cox3, nad1, nad2, nad3, nad4, two copies of nad4L, two copies of nad5, nad6, atp6, atp8, and atp9. There were two ribosomal protein-encoding genes (rnl and rps3) and the DNA polymerase gene (dpo) in the genome. It also had 53 protein-coding genes with unknown function in addition to 21 and 4 homologous to LAGLIDADG and GIY homing endonucleases, respectively (**Figure 4B**).

### Kegg and GO analysis

**Figure 5:**
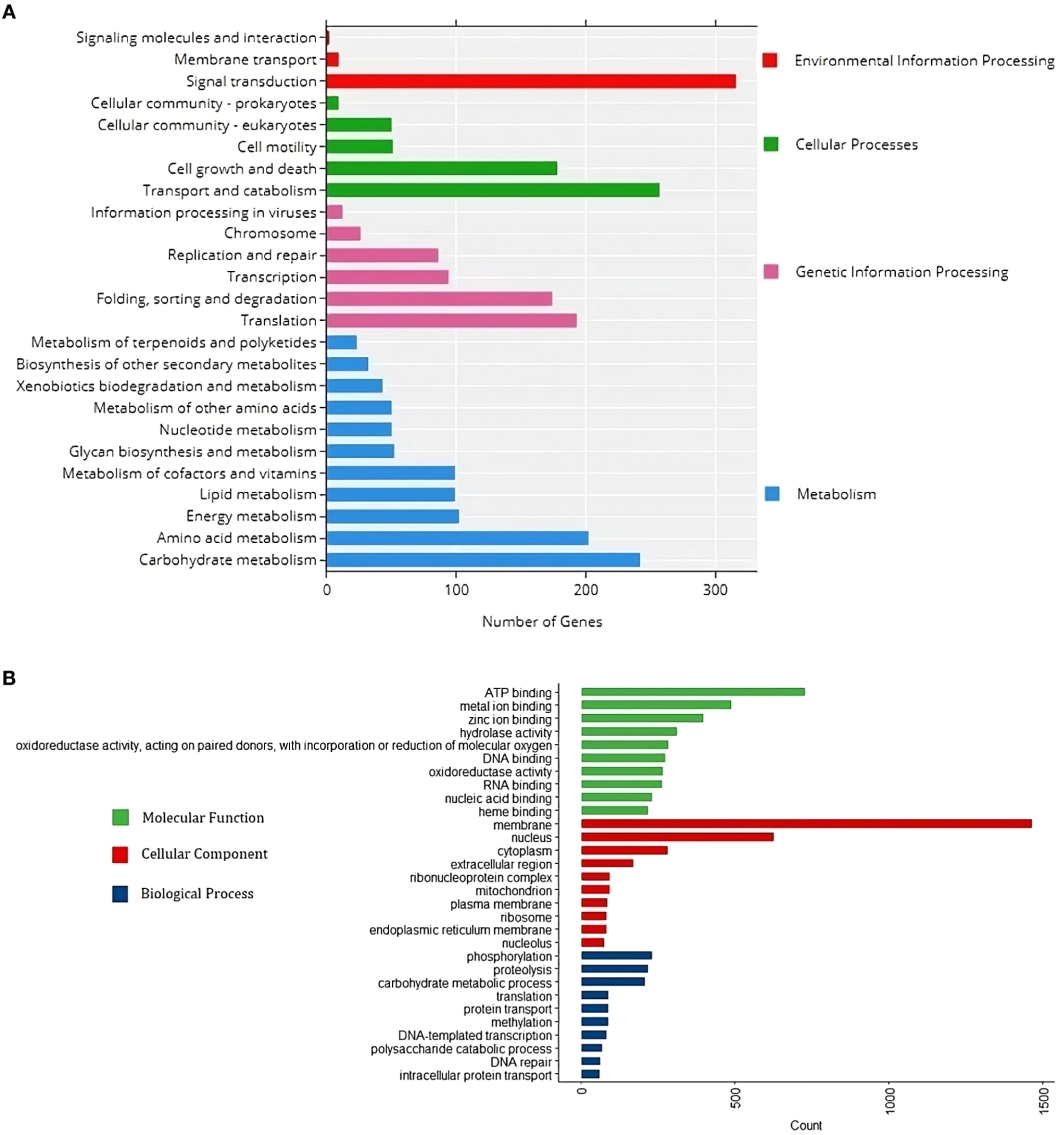
Functional annotation of the *R. solani* (RAP2) genome infecting Barnyard Millet. **(A)** KEGG annotation chart at Level 2. The horizontal x-axis is the number of genes, the vertical y-axis represents the name of the Level 2 pathway, and the length of horizontal bars designates the number of genes annotated to the Level 2 pathway. The legend corresponds to the Level 1 hierarchy to which Level 2 pathways belong. **(B)** GO functional annotation classification statistics plot. The horizontal x-axis is the number of genes, the vertical y-axis consists of a set of ontology terms belonging to the gene ontology domains, molecular function, cellular components and biological process.

Protein sequences correlated with KEGG pathway maps reveal that “Carbohydrate metabolism” has the highest gene enrichment in the “Metabolism” category, while “Translation” has the highest gene enrichment in the “Genetic information and processing” category. “Transport and Catabolism” and “Signal Transduction” pathways have the highest gene enrichment in “Cellular processes” and “Environmental information processing” categories, respectively (**Figure 5A**). The UniprotR package’s gene ontology analysis shows that the terms “phosphorylation,” “membrane,” and “ATP binding” have the highest gene enrichment in the “biological process”, “cellular component”, and “molecular function” domains, respectively (**Figure 5B**).

### Secretome analysis

**Figure 6:**
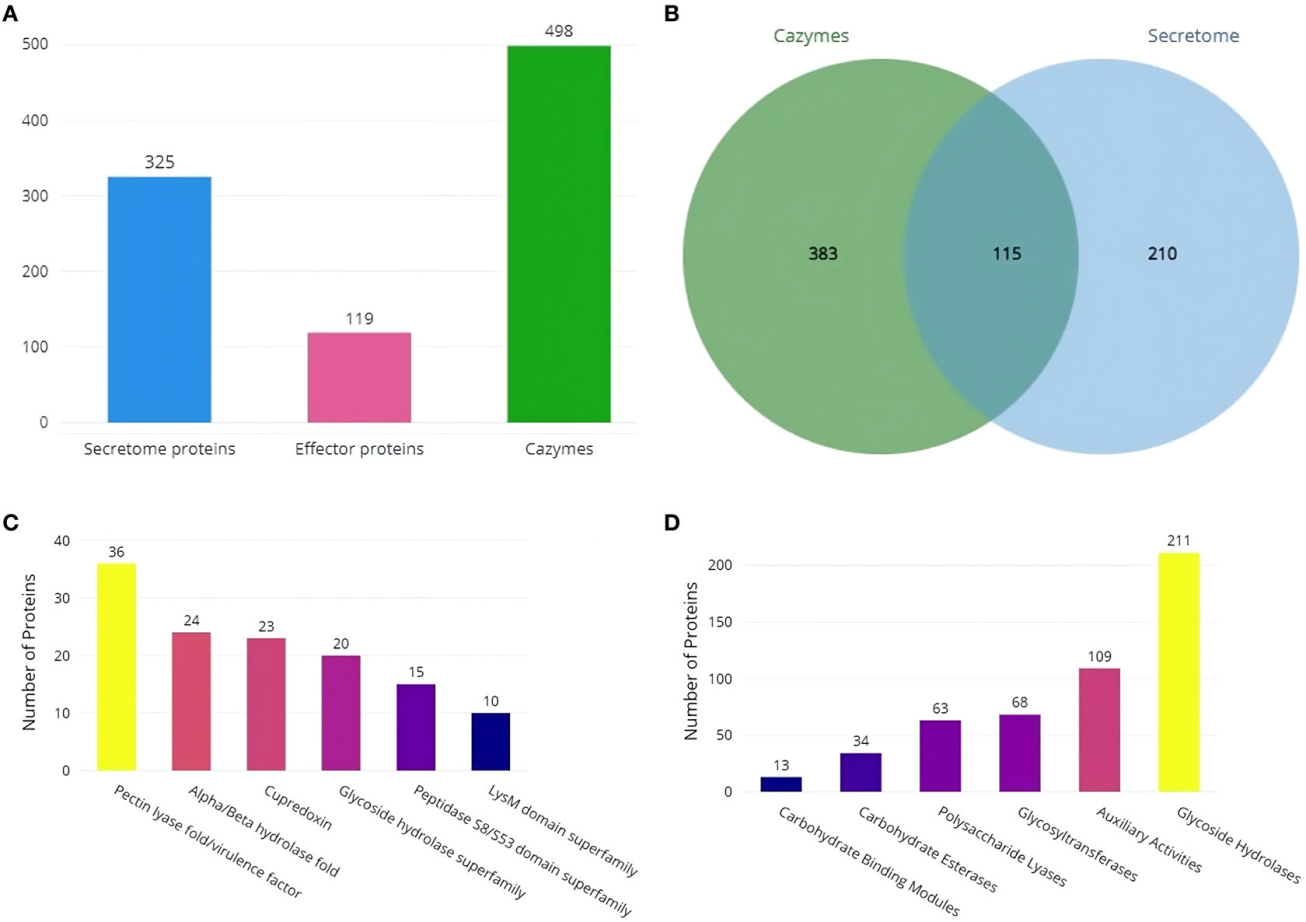
Secretome and Cazyme analysis of RAP2 genome **(A)** Predicted number of Secretome, Effector and Cazyme proteins **(B)** Number of proteins shared by both the dbCAN3 and Secretool predictions **(C)** SUPERFAMILY annotations of secretome proteins and **(D)** Distribution of Cazymes classes predicted in the RAP2 genome.

Secretool predicted 325 proteins from the assembled RAP2 genome, with 119 predicted by the EffectorP tool as proteins with signal peptides for secretion (**Figure 6A**). Most secretome proteins belong to the pectin lyase fold/virulence factor superfamily, followed by the Alpha/Beta hydrolase fold, Cupredoxin, Glycoside hydrolase, Peptidase S8/S53 domain, and LysM domain superfamilies (**Figure 6C**). dbCAN3 predicted 498 proteins as cazymes, with 115 proteins being shared with secretool predictions (**Figure 6B**). Predicted cazymes belonged to the Glycoside Hydrolases class, followed by Auxiliary Activities, Glycosyltransferases, Polysaccharide Lyases, Carbohydrate Esterases, and Carbohydrate-Binding Modules classes (**Figure 6D**).

### Comparative genomics analysis

**Figure 7:**
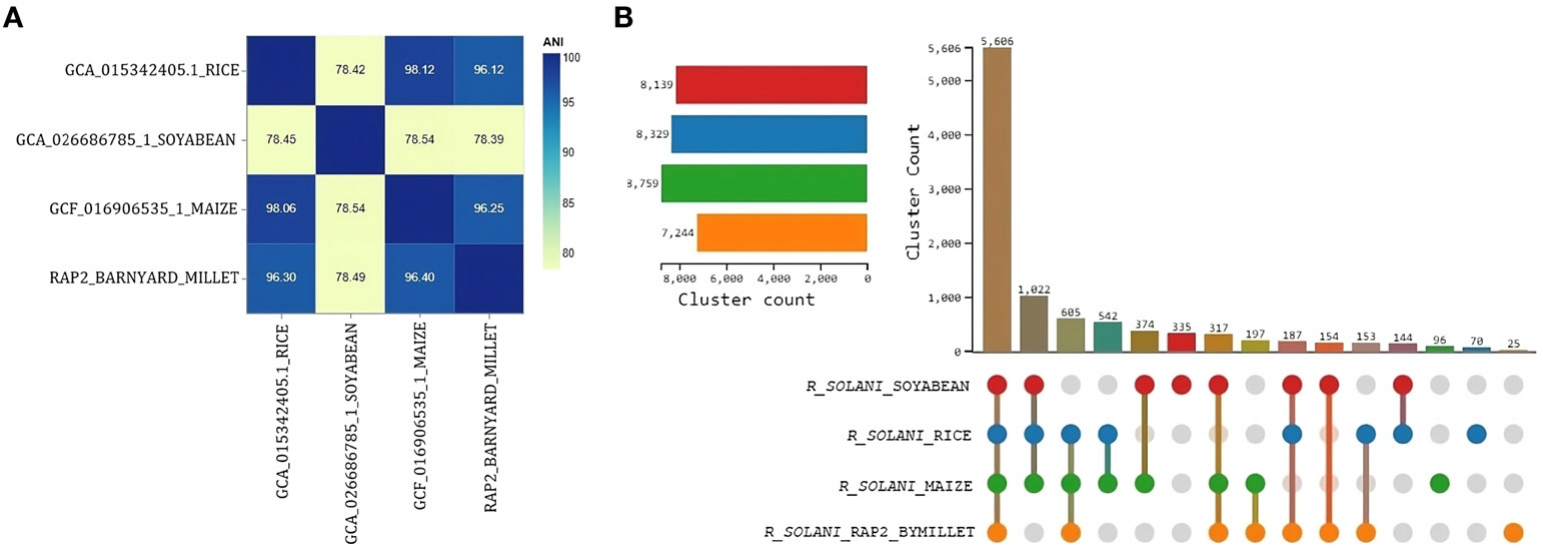
Comparative genomics analysis of *R. solani* genomes **(A)** Average Nucleotide Identity measure among the *R. solani* genomes **(B)** UpSet graph illustrating the distribution of orthologous clusters among different *R. solani* genomes.

All four *Rhizoctonia solani* genomes contained more than 84% complete and single-copy BUSCOs, according to expected gene content analysis. The majority were present in maize, followed by barnyard millet, soybeans, and rice. When compared to other genome assemblies, the RAP2 genome assembly exhibited fewer missing BUSCOs (**Supplementary Figure S2A**). When compared to the 260 BUSCOs in the eukaryota dataset, the RAP2 genome lacked 6 orthologs, of which 4 were absent from all other *Rhizoctonia solani* genomes. Only the RAP2 genome, however, was lacking a single BUSCO (**Supplementary Figure S2B**). The RAP2 genome and the *Rhizoctonia solani* genomes that infect rice and maize had ANI values greater than 96, indicating species-level clustering; in contrast, the ANI value with the *Rhizoctonia solani* genome that infects soybean was less than 96, indicating further genome divergence (**Figure 7A**).

OrthoVenn3 analysis of RAP2 protein sequences found 8 clusters with 5606 proteins shared by all fungal genomes. A singleton cluster with 25 proteins was specific to the RAP2 genome (**Figure 7B**). Functional Gene Ontology analysis of shared proteins revealed that GO:0008150 (biological process), GO:0016491 (oxidoreductase activity), and GO:0016020 (membrane) were the top three enriched terms (**Supplementary Figure S3**). Whereas for the singleton cluster containing 25 proteins of the RAP2 genome, the enriched GO terms were GO:0019277 (UDP-N-acetylgalactosamine biosynthetic process), GO:0015940 (pantothenate biosynthetic process), GO:0009723 (response to ethylene), and GO:0006782 (protoporphyrinogen IX biosynthetic process) (**Table 5**).

**Table 5:** OrthoVenn3 GO enrichment of the proteins clustered specific to RAP2 genome.

GO ID	Namespace	Name	Count	p-value
GO:0019277	biological_process	UDP-N-acetylgalactosamine biosynthetic process	2	2.2E-05
GO:0015940	biological_process	pantothenate biosynthetic process	2	0.001
GO:0009723	biological_process	response to ethylene	2	0.002
GO:0006782	biological_process	protoporphyrinogen IX biosynthetic process	2	0.003

Two proteins were identified as cutinase proteins in the RAP2 genome, and one protein had an intact cutinase domain. Additionally, cutinase proteins were retrieved from the genomes of *Rhizoctonia solani*, infecting rice, soybeans, and maize. Multiple sequence alignment of cutinase proteins indicate the presence of the GYSKG motif unique to *Rhizoctonia solani* cutinases. Out of 166 amino acid bases containing cutinase domain, 112 amino acid bases were conserved, with a specific 28-base deletion observed in the cutinase domain in the RAP2 genome (**Supplementary Figure S4**).

## Discussion

This study has provided a comprehensive exploration of *R. solani*, the pathogenic fungus that infects barnyard millet. As a formidable soil-borne pathogen, *R. solani* is notorious for causing sheath blight disease across a range of Poaceae crops. The disease name “banded sheath blight” is due to its unique banded appearance, which is characterized by ovoid to irregular, light gray to dark brown lesions on the lower leaf sheath that grow rapidly and coalesce to cover vast parts of the sheath and leaf lamina. Significant variations were observed in the morphology, sclerotial structure, and mycelial characteristics of the *Rhizoctonia* isolates. Sclerotia exhibited either smooth or rough textures and varied in color from dark brown to yellow, while the colonies ranged in appearance from yellow to brownish-yellow. Similar morphological heterogeneity has been reported in previous studies of *Rhizoctonia* isolates (Srinivas et al., 2007; Moni et al., 2016). Notably, each isolate showed distinct differences in virulence when inoculated onto the susceptible barnyard millet cultivar LDR-1. Virulence assessments revealed that the RAP2 strain exhibited particularly high virulence, leading to its selection for whole-genome sequencing.

We have successfully sequenced, assembled and annotated the genome of the most virulent *R. solani* strain, RAP2, to enhance our understanding of the host-pathogen interaction with barnyard millet. The RAP2 genome comprises 4162 contigs with a total size of 43.63 megabases and an N50 contig length of 1,854,826 bp. The N50 value is represented by 8 contigs (L50), while the N90 contig length was 3779 bp, with the L90 value encompassing over 664 contigs.

In comparison, the model reference strain (GCF_016906535.1) in the NCBI RefSq database has a genome size of 40.85 megabases with 17 contigs, which is less smaller than the RAP2 genome. The observed variation in genome size may reflect biological adaptations that enable the strain to interact with new host species by enhancing its ability to recognize novel hosts, produce toxins, and evade plant immunity (Kemen, 2012; Möller and Stukenbrock, 2017). Alternatively, the variation could be attributed to technical factors, such as increased fragmentation of the RAP2 genome. The RAP2 mitochondrial genome exhibits 99.9% identity with over 93% coverage of the AG1-IA strain mitogenome of *R. solani*, which affects maize and rice. Despite this high identity, the RAP2 mitogenome shows lower coverage percentages—51% compared to the lettuce-infecting AG1-IB strain, 47% compared to the AG1-IC strain known to infect cabbage and soybeans (Lin et al., 2021), and 27% compared to the AH4 strain, which infects potatoes.

Repetitive DNA is a crucial component of eukaryotic genomes, with transposable elements capable of relocating within the genome (Muñoz-López and García-Pérez, 2010). Variations in the abundance of these can significantly impact genomic architecture (Möller and Stukenbrock, 2017), potentially leading to genomic expansion and enhancing the pathogen’s ability to counteract host defenses (Muszewska et al., 2019). The RAP2 genome, repetitive DNA constitutes 10.95% of the total, predominantly comprising interspersed elements. Of these, approximately 46% are retroelements, 8% are DNA transposons, and the remaining 46% are unidentified. The retroelements are primarily Ty3-retrotransposons, while MULE-MuDR elements are the most common among the DNA transposons. Ty3-retrotransposons are known to facilitate genome expansion and stress-induced adaptation (Wacker et al., 2023). During plant infection, de-repression of Ty3-retrotransposons occurs, and subsequent silencing of these elements can lead to reduced virulence (Fouché et al., 2020). Additionally, MULE DNA transposons have a propensity to integrate into or near gene regions, which can be highly mutagenic (Dupeyron et al., 2019).

The GO annotation of RAP2 proteins highlights “phosphorylation” as the most significantly enriched “biological process” domain. This enrichment underscores the critical role of phosphorylation-dependent signaling pathways in the growth of appressoria, a process intimately associated with biotrophic and hemibiotrophic fungal infections (Cruz-Mireles et al., 2024). Additionally, within the cellular component domain, “membrane” emerged as the most enriched category, while “ATP binding” dominated the molecular function domain. Given that lipids and fatty acids are fundamental constituents of biological membranes, including mitochondrial, cellular, and plasma membranes, the stability of the fungal pathogen membranes may be influenced by ATP-binding proteins involved in phospholipid biosynthesis (Zhang et al., 2022). KEGG pathway analysis further revealed significant enrichment in carbohydrate metabolism and signal transduction pathways. Carbohydrate-containing compounds secreted by plant pathogens often act as toxins, eliciting disease symptoms in plants. Pathogen enzymes, which degrade plant structural polysaccharides, are crucial to this process (Kosuge, 1981). Furthermore, the differentiation and virulence of fungal pathogens, including those affecting plants and animals, are regulated by two conserved signal transduction cascades: the MAP kinase signaling pathway and the cAMP-PKA signaling pathway, both of which are essential for appressorium development and fungal pathogenicity (Lengeler et al., 2000).

The ability of pathogenic fungi to infect the hosts is fundamentally reliant on their secretion of specific proteins. Upon encountering the plant surface, these fungi initially contact the cuticle and secrete cutinase, an enzyme that catalyzes the hydrolysis of cutin polymers present on plant surfaces. Cutinase2, in particular, is critically overexpressed during both the development and maturation of the appressorium, a key stage in fungal infection. Additionally, upon breaching the plant epidermis, pathogenic fungi release a suite of enzymes that degrade pectin and cellulose in the plant cell wall. This enzymatic activity facilitates the acquisition of polysaccharides, which are essential for the fungus growth and reproductive processes (Jia et al., 2023).

In the RAP2 genome, the majority of secretory proteins are categorized within the pectin lyase fold/virulence factor superfamily. CAZymes are pivotal in degrading break down plant-cell-wall polymers, thus liberating saccharides that serve as carbon sources for the fungi (Hage and Rosso, 2021). Among these, glycoside hydrolases represent the most extensive class of CAZymes, responsible for hydrolyzing glycosidic bonds. These enzymes are crucial for host colonization and play a significant role in activating the host’s immune responses (Bradley et al., 2022). Notably, the RAP2 genome is distinguished by its high abundance of CAZymes classified as glycoside hydrolases.

Comparative analysis of the RAP2 genome with those genomes of *R. solani* infecting maize and rice, both of which are the members of the Poaceae family, reveals an average nucleotide identity (ANI) exceeding 96%, indicating a high level of genomic similarity and suggesting close clustering at the species level. In contrast, the ANI drops below 96% when comparing RAP2 with the *R. solani* genome, which infects soybean, reflecting a greater degree of divergence in genomic similarity. Furthermore, orthologous clustering of RAP2 protein sequences with those from maize, rice, and soybean identifies 5606 proteins that are conserved across all these genomes. GO analysis of 25 proteins unique to the RAP2 genome reveals significant enrichment in pathways related to ethylene response and the biosynthesis of pantothenate, protoporphyrinogen IX, and UDP-N-acetylgalactosamine. Ethylene is known to trigger spore germination and subsequent infection in host plants by plant pathogenic fungi (Ren et al., 2022). Pantothenic acid biosynthesis is crucial for maintaining iron homeostasis and contributes to pathogenicity in *Aspergillus fumigatus* (Dietl et al., 2018). The synthesis of heme, essential for peroxidase activity, involves the conversion of coproporphyrinogen III to protoporphyrinogen IX, with peroxidases playing a pivotal role in resisting oxidative stress (Park et al., 2023). Additionally, UDP-N-acetylgalactosamine is involved in several pathogenic processes, such as biofilm formation, immune evasion, and antifungal resistance (Le and Sheppard, 2023).

The cutinase protein plays a crucial role in the infection process by catalyzing the degradation of cutin. Analysis of multiple sequence alignments comparing the cutinase proteins from the RAP2 genome with those from maize, rice, and soybean identified a distinctive 28-bp deletion in a highly conserved region of the RAP2 cutinase protein. This unique deletion suggests a potential alteration in the protein’s function. We propose that this 28-bp deletion in the RAP2 cutinase domain may either lead to a loss of enzymatic activity or confer the pathogen with the capability to infect barnyard millet while evading host defense mechanisms.

This study, which delves into the genomic analysis of *R. solani* infecting barnyard millet, lays a crucial foundation for developing targeted control measures by providing comprehensive genome and protein sequence data. A more profound understanding of *R. solani* effector biology will enhance our capacity to counteract its multifunctional effectors, which pose significant challenges to the immune responses of barnyard millet. An in-depth investigation of the genes associated with pathogenicity could unveil new strategies for management, particularly through the recent identification of effector proteins that open avenues for host-induced gene silencing (HIGS) approaches to combat this disease (Yu et al., 2023; Sunani et al., 2024). Nonetheless, conducting functional assays to validate the identified effector proteins and carbohydrate-active enzymes (CAZymes) is imperative.

## Conclusion

Bsb disease in cereals is caused by the soil-born basidiomycetes fungal pathogen *R. solani*. Throughout their evolutionary process, plant pathogenic fungi often survive by jumping from one host to another. Although they are recognized as hardy crops, millets are now under biotic stress. For instance, an observation of *R. solani* infecting barnyard millet is made in this study. Through virulence studies, the most virulent strain was identified and designated as RAP2. Although the genetic and genomic resources of the *R. solani* strains that infect commercial crops such as rice, maize, and soybean are accessible, this study provides the scientific community with pathogenomic insights into the *R. solani* infecting barnyard millet, allowing researchers to gain a deeper understanding of the pathogen’s genetic makeup.

We have sequenced the RAP2 strain’s reasonably high-quality genome and performed *de novo* assembly, annotation, and functional genomic research. The genome is 43.63 MB in size, and its mitochondrial genome is 99.9% identical to the *R. solani* AG1-IA strain. The repetitive genome landscape primarily comprises interspersed elements. The RAP2 genome exhibits enrichment in pathways like signal transduction and carbohydrate metabolism. The RAP2 genome’s secretory proteins are members of the pectin lyase fold/virulence factor superfamily, which is responsible for breaking down the pectin in cell walls that is necessary for the pathogen’s growth and reproduction. Glycoside hydrolases are the largest class of CAZymes in the RAP2 genome and are known to promote host colonization.

A comparative genome analysis of RAP2 with *R. solani* strains infecting both the Poaceae (rice and maize) and Fabaceae (soybean) families reveals that the RAP2 genome clusters together at the species level with the Poaceae family strain and diverges further in terms of genomic similarity with the Fabaceae family strain. A unique 28-bp deletion in a highly conserved region has been observed in the cutinase protein of the RAP2 genome. These findings provide significant new insights into the genetic composition, virulence factors, and molecular mechanisms that *R. solani* strain RAP2 might employ to adapt to barnyard millet and may hold great promise for the development of precise control measures for *R. solani* causing Bsb disease in millets.

## Data Availability

The *R. solani* strain RAP2 Whole-Genome shotgun experiment has been deposited in the NCBI SRA database under the BioProject ID PRJNA1112772 and the BioSample ID SAMN41434317. The Whole genome assembly of *Rhizoctonia solani* (RAP2 strain) was deposited in the NCBI repository under the accession number JBEGIX000000000, with BioProject ID number PRJNA1112772 and BioSample ID number SAMN41434317.
