# Supplementary material for: Virulence perspective genomic research unlocks the secrets of Rhizoctonia solani associated with banded sheath blight in Barnyard Millet (Echinochloa frumentacea)
Source: Front Plant Sci. 2024 Oct 28;15:1457912. doi: 10.3389/fpls.2024.1457912 (PMC11551851; doi:10.3389/fpls.2024.1457912)
Supplement: Supplementary file 1 [file DataSheet1.docx]

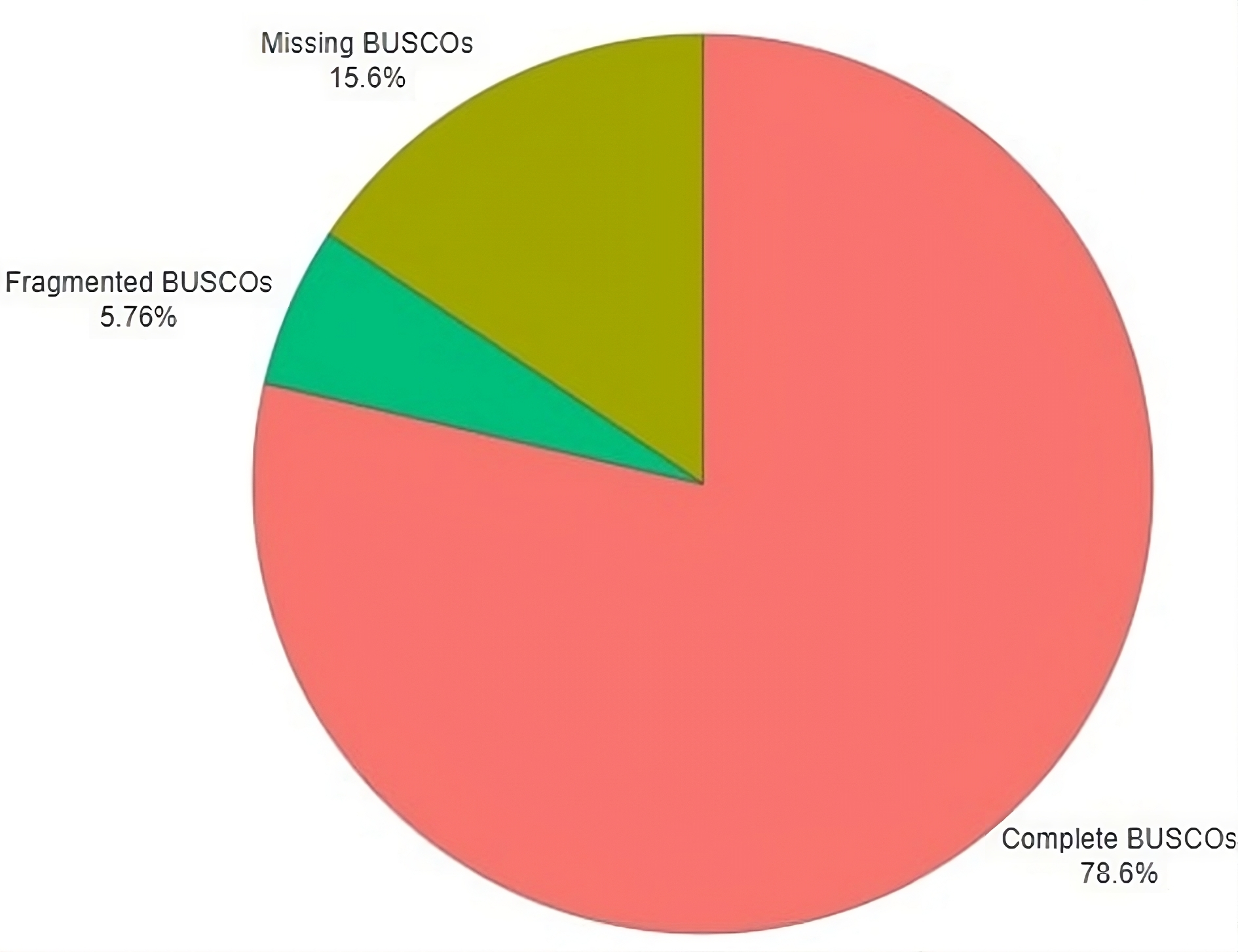


**Figure S1:** BUSCO assembly completeness measure of RAP2 genome against agaricomycetes dataset of 2898 conserved buscos
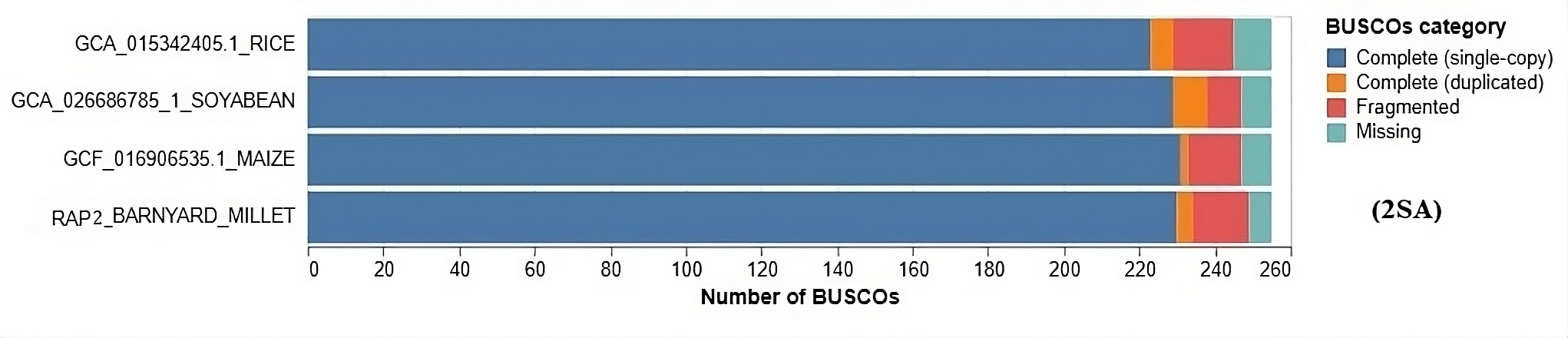


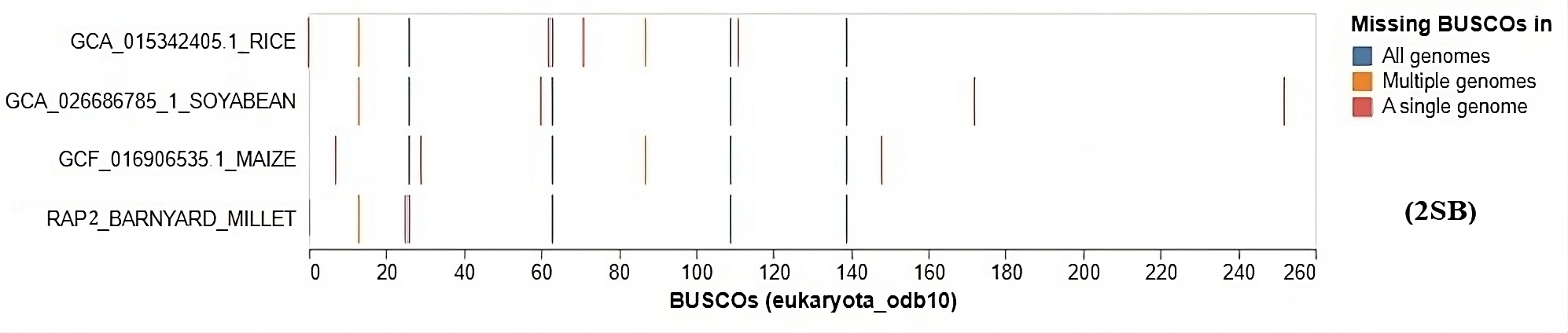


**Figure S2:** Assembly completeness measure of compared genomes against eukaryota dataset of 260 buscos (A) Summary of complete BUSCOs (B) Summary of missing BUSCOs


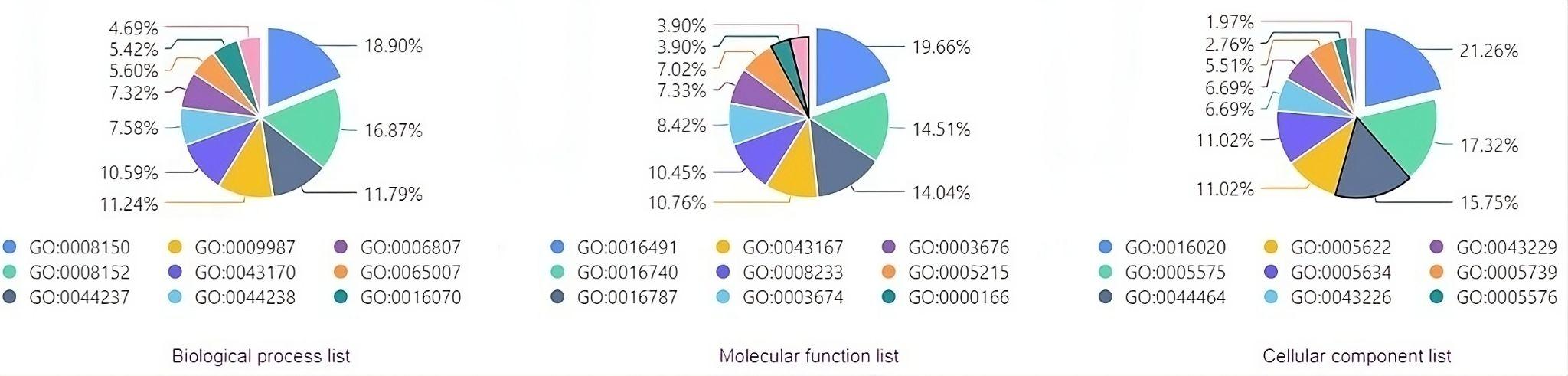


**Figure S3:** GO analysis of shared ortholog proteins present in the *R solani* genomes


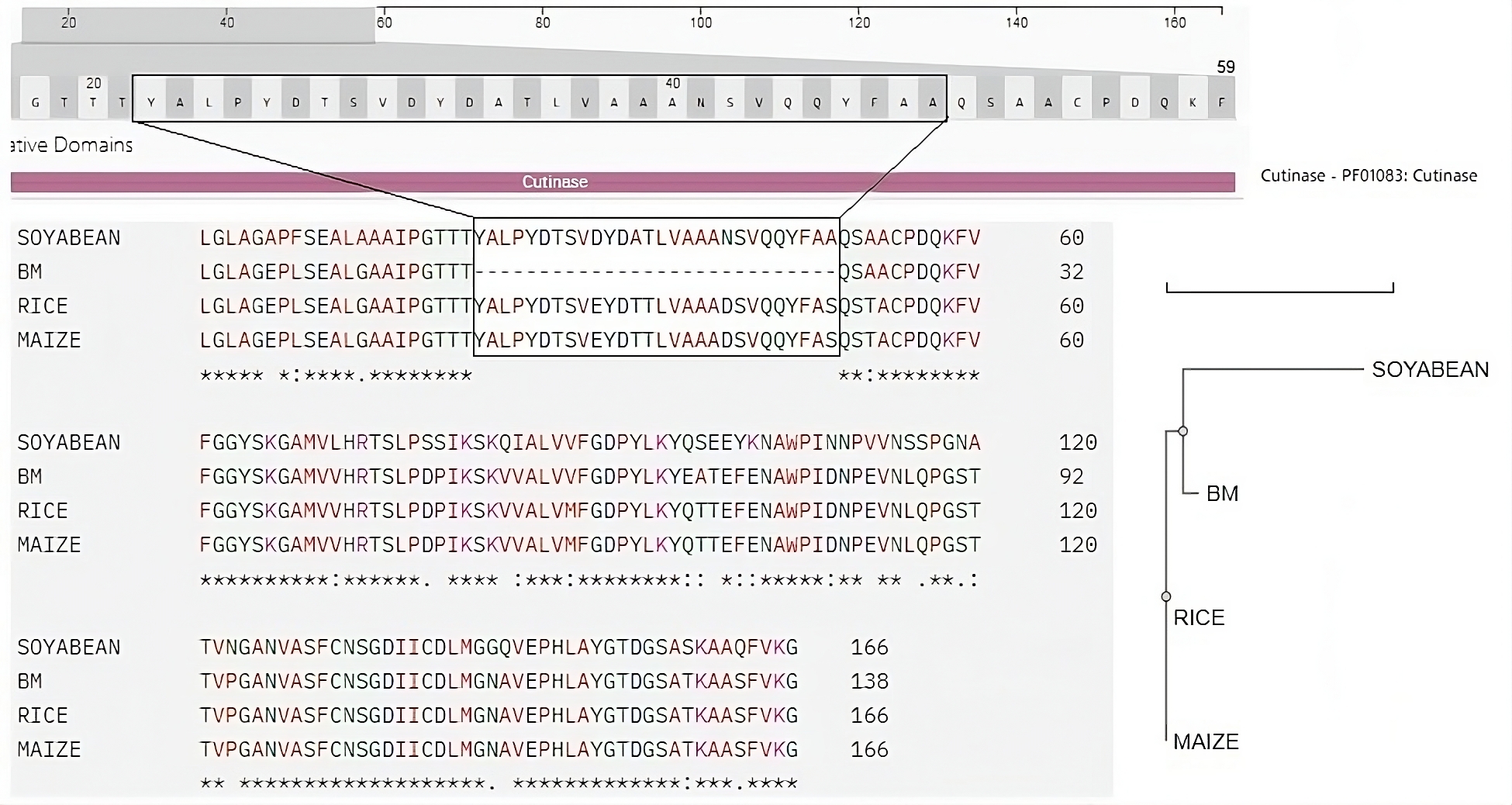


**Figure S4:** Multiple sequence alignment of cutinase proteins present in the *R solani* genomes

**Table S1**: Illumina raw read and filtered read statistics of the RAP2 genome

| **RAP2 (Read Quality)** | **Total Sequences** | **Sequence length** | **Percent (%) of sequences** | **GC%** |
| --- | --- | --- | --- | --- |
| Raw Reads | 18807680 | 159 | 100.00 | 47 |
| Filtered Reads | 18696879 | 20-144 | 99.41 | 47 |

**Table S2**: RAP2 Genome Statistics generated by QUAST on the assembly processes including genome-guided correction, scaffolding, repetitive contig filtering, and retention of contigs ≥500 bp, compared to AG-1 IA strain genome

| **Assembly metric** | ***de novo* assembly** | **RagTag assembly** | **Funannotate (final RAP2 assembly)** | **GCF_016906535.1 (AG-1 IA)** |
| --- | --- | --- | --- | --- |
| Assembly size (bp) | 65331303 | 67588503 | 43638810 | 40856322 |
| Total contigs | 205572 | 183000 | 4162 | 17 |
| Largest contig (bp) | 99607 | 3788558 | 3788558 | 3756861 |
| N50 (bp) | 3958 | 2056884 | 1854826 | 2303118 |
| N90 (bp) | 749 | 3499 | 3779 | 2094219 |
| L50 | 2531 | 8 | 8 | 7 |
| L90 | 12341 | 733 | 664 | 14 |
| # N's per 100 kbp | 227.04 | 5343.79 | 5376.79 | 0.00 |
